# *“I always know she cannot betray me*.*”* Disclosure of abortion and methods of abortion used in informal settlements in Nairobi, Kenya

**DOI:** 10.1371/journal.pgph.0003252

**Published:** 2024-07-17

**Authors:** Ramatou Ouedraogo, Shelmith Wanjiru, Moussa L. Zan, Clementine Rossier, Onikepe Owolabi, Sherine Athero, Clement Oduor, Martin Bangha, the N-MAS team

**Affiliations:** 1 African Population and Health Research Center, Nairobi, Kenya; 2 Graduate Institute of International and Development Studies, Geneva, Switzerland; 3 Institut Supérieur des Sciences de la Population, Ouagadougou, Burkina Faso; 4 University of Geneva, Geneva, Switzerland; 5 Guttmacher Institute, New York, New York, United States of America; Tata Institute of Social Sciences, INDIA

## Abstract

Despite abortion being stigmatized and legally restricted in Kenya, women still disclose their abortions within their network. Evidence has shown how stigma can influence and regulate individual abortion disclosure decisions and behaviors. This paper seeks to understand why and how women make the decisions to disclose their abortion and the associated methods used. The data are from a qualitative formative study and a respondent-driven sampling survey conducted between 2020 and 2021 in two informal settlements in Nairobi, Kenya. The data were analyzed using a descriptive analysis approach for the quantitative data, and thematic analysis for the qualitative data. Our findings reveal that information sharing about abortion is enclosed in a social dynamic of secrecy. This dynamic contributes to making abortion a secret that respondents decided to share with confidants in 81% of the abortion cases. These confidants include intimate relationships such as trusted friends (62%), followed by female relatives. Information was shared in many cases either to get support (i.e. method to use), or because participants had close ties with the confidants. Regarding the methods used, unidentified pills were the most used regardless of the confidant; followed by traditional methods especially among those who sought help with their mothers/aunts/grandmothers (33%), while Medical Abortion and Manual Vacuum Aspiration were rarely used, mostly by those who confided in friends or sisters/cousins. Our findings show that the disclosure of abortion is a complex process embedded in existing codes regarding the circulation of information on sensitive issues and “help” seeking. Our findings show that the need for information on safe abortion and lack of financial resources frequently empowers them to overcome the fear of stigma and disclose their abortion. However, this often resulted in use of unsafe procedures. The findings suggest the need for strengthening the circulation of information on safe methods within communities, using community champions and intermediaries to increase the likelihood of women being directed through safe methods to enhance their use.

## Introduction

Unintended pregnancy is a common experience for many women in sub-Saharan Africa (SSA). The region has one of the highest rates of unintended pregnancies in the world, at 91 per 1000 women aged 15–49, compared to 35 per 1000 women in Europe and North America [1]. A report by the World Health Organization (WHO) estimates that over 60% of these pregnancies are aborted and that 45% of these abortions are unsafe [2]. In Kenya, there were 2,380,000 pregnancies per year between 2015 and 2019, of which 1,450,000 were unintended [1]. While unintended pregnancies are recurrent events that women face in SSA countries and women are likely to consider pregnancy termination as an option to avoid an unintended birth, the restrictive legal framework and prevailing stigma often shape the abortion pathways and outcomes in the region [3]. In Kenya, for instance, induced abortion is legally restricted, and permitted only to save the life and/or preserve the physical health of a pregnant woman [4]. Additionally, girls and women who have abortions face social stigma [5–8]. Together, the legal restriction of abortion and the social and religious reprobation create a stigmatizing context that influences access to safe services, leading to numerous unsafe abortions. Of the unintended pregnancies reported per year between 2015 and 2019 in Kenya, close to half (551,000) resulted in abortions that were unsafe [1].

Stigma related to abortion is a result of specific gender norms that value women’s self-restraint from sexuality and venerate their role as mothers [9]. Abortion implies a variety of transgressions, including participating in sexual activities without a desire to reproduce and a lack of desire for marriage and, ultimately, lack of desire for motherhood [10]. Erving Goffman [11] categorized stigma surrounding abortion into two of the three categories of concealable stigma: tribal stigma (where girls, wives, and mothers are classed into two tribes of "good" and "bad" depending on their behavior) and blemish of character (degradation of a woman’s moral character). In this regard, women from communities where norms condemn and reproach such practices will tend to conceal their abortions [12] and be selective in terms of whom they reveal the information to [10].

It is important to note that stigma can be perceived, experienced, and internalized [13]. Concerning abortion, authors posit that “perceived stigma” refers to the recognition of other people’s undesirable attitudes regarding her experience with abortion, including the expectation that these attitudes may end up in real discrimination [14]. As a result, women may associate these negative attitudes with their self-image and internalize these feelings of guilt and shame [14]. Abortion stigma exists in contexts with both liberal and restrictive abortion legislation, but it is more prominent in the latter [15]. Legal restrictions have hampered access to safe abortion and post-abortion services because disclosure might result in associated stigma and criminal punishment for the woman seeking the abortion [16]. Increasingly, a body of research has been accumulating linking the overall secrecy and silence around abortion to fear of related stigma [16].

However, despite abortion being a stigmatized practice and reducing the likelihood of information disclosure, women still disclose information on their abortions within their networks [17]. Evidence has shown how stigma can influence and regulate individual abortion disclosure decisions and behaviors [17, 18]. Disclosure behaviors, particularly to whom the abortions are disclosed, can have a huge impact on the women’s decision-making and care-seeking pathway. According to Coast and Murray [19], trusted advice from others played a significant role in women’s abortion trajectories, with the quality of the relationships, expectations, and ideals within them also being crucial factors. The person (s) to whom women reveal their information may have an impact on whether or not they decide to have an abortion, may be a source of (mis) information, may expose them to unsafe procedure or give access to abortion-related care [20].

Previous studies have shown that disclosure tends to vary depending on the accessibility of abortion, the legal context, and the level of (fear of) stigma. According to previous research, individuals frequently consult with a large number of people in their network before obtaining abortion services [21]. Studies conducted in Kenya show how accessing safe abortion services might be impeded by a number of factors, such as high costs and the fear of stigma [7]. To overcome these barriers, girls and women often turn to friends and relatives for recommendations on anonymous providers, such as traditional birth attendants, informal providers, or local drug vendors and pharmacists, who in turn, frequently serve referrals from past patients [22]. However, a systematic review on disclosure among abortion-seeking women in low- and middle-income countries identified further gaps, reporting that studies done on this subject mostly fail to explicitly describe the degree of disclosure, the kind of information communicated, the precise circumstances surrounding disclosure, the amount of trust and secrecy, and the person talked to [23].

Consequently and to understand this phenomenon, this study adopted both quantitative and qualitative approaches to examine dynamics around abortion secrecy, concealment, and disclosure and how these influence access to safe abortion services. Using the lens of abortion stigma, secrecy, and support networks, this manuscript aims to understand why and how women make the decisions to disclose the information regarding their unintended pregnancy and/or eventual termination, to whom the information is shared with and the outcomes of the disclosure in terms of methods used. We examine the dynamics embedded in girls’ and women’s networks, focusing on the mechanism that motivates them to share secrets. George Simmel [24] contends in his sociology of secrecy that the dynamics of secrecy are greatly influenced by the structure of social networks. He argued that the flow of secrets among these networks is dynamic and constantly evolving. He also emphasized the importance of trust in the flow of secrecy, suggesting that individuals are more willing to disclose secrets with those they trust and that trust is often established through the sharing of secrets [24, 25]. Aside from trust, self-interest can also play a role in the decision to disclose a secret, in situations where they anticipate potential benefits, such as financial or emotional support [24]. Additionally, Zempleni [25] explained that secrets are often disclosed to a confidant, who may play many roles, including therapist, supporter, or close friend. People are often more inclined to share secrets with others if they believe that they are in similar life situations or have had similar experiences in the past [24, 25].

Using qualitative and quantitative data collected in Kenya, we attempt to tease out the hidden social rules surrounding information sharing, particularly on sensitive discussions such as access to abortion services to pinpoint the rationale and the circumstances of disclosure (i.e. information being disclosed voluntarily versus information being leaked for a variety of reasons). Additionally, we investigate the interplay of factors like cost of services and stigma on access to safe abortion services, as well as the role disclosure and social networks play in facilitating access to abortion services.

## Methods

### Study site and population

This paper draws from a mixed-methods cross-sectional study involving a formative qualitative study conducted between October 1 to 17, 2020 and a Respondent-Driven Sampling (RDS) survey conducted between August 24 and November 11, 2021 in Korogocho and Viwandani informal settlements in Nairobi, Kenya. The data were collected as part of a larger study, supported by the World Health Organization (WHO), which sought to describe the safety of abortion at the population level using network-based survey approaches in Burkina Faso and Kenya [26]. The study targeted women of reproductive age (15–49 years old), who have lived in the study community for at least six months preceding the survey and had an abortion experience within the past three years.

### Sampling and data collection approach

The data were generated in two phases. The first phase involved the formative qualitative study and aimed at exploring the dynamics around secrecy and the sharing of abortion information in women networks. The findings were meant to inform the feasibility of the RDS (which heavily draws on social network) and inform the design of the questionnaire. The formative study was followed by the RDS survey one year later.

#### Formative qualitative study

The qualitative study team consisted of two research assistants who had qualifications in social sciences, and had prior experience in qualitative data collection and working in informal settlements. The research assistants were trained for five days, and the training covered a value clarification and attitude transformation (VCAT) session, the objectives of the study, the tools and data collection techniques, research ethics, practical role plays, and piloting of the tools.

The formative qualitative study target women of reproductive who have undergone abortion. The participants were purposively selected to ensure diversity (i.e. age, marital status, occupation). For the recruitment of the participants, research assistants actively worked with a community mobilizer (community health volunteer -CHV to help identify the first participants and complemented the process with snowballing approach (participant linking the study team to other women they knew have had experienced abortion. Upon being introduced to the study and the study team, the community mobilizer identified potential women who met the inclusion criteria and recommended those who were interested in participating in the "women’s health study"- as the study was referred to in the community, to avoid the possibility of negative publicity and stigmatization. The recommended women who expressed interest in participating in the study were directed to the study’s centralized interview venue, where they were screened for eligibility before being interviewed. The interviews were conducted using a semi-structured interview guide, mostly in Swahili, with very few in English, and recorded. The interview focused on women’s support networks, the differentiation between secrets and important things, sharing abortion secrets, reasons for sharing, rationale for selecting confidants, and abortion decision-making and care-seeking pathways.

#### Respondent driven sampling

RDS is a sampling approach used to study hard-to-reach populations and hidden practices through peer-to-peer recruitment [27, 28]. Though recent, the use of RDS in abortion studies has proven to be appropriate and effective in generating representative samples of women who have had abortions [29, 30]. For this study, the RDS targeted women who had abortions in the past three years and were living in the study sites (Korogocho and Viwandani). In the two sites, we used the population distribution to design a target subsample for each of the sub-areas. Hence, 70% of the sample was to be collected in Korogocho and 30% in Viwandani.

RDS data were collected by four research assistants who were trained on the study objective, approach, and tools. For the identification of the participants, we used the CHVs as entry point as for the qualitative study. The research assistants were linked to two CHVs who were responsible for the identification, mobilization, and referral of primary seeds. "Seeds" refers to the initial participants with experience in the target event and selected to initiate the sampling process, and subsequently help in identifying other respondents. We had in total eight seeds in Korogocho and five in Viwandani. We carefully selected the seeds to ensure diversity in their sociodemographic characteristics (i.e., younger unmarried versus older divorced or separated, more or less educated, diverse occupations). The seeds were, then, screened for eligibility, and those who were found to be eligible and consented to participate in the study were interviewed. The successfully interviewed seeds and subsequent recruits were taken through the study’s recruitment procedures and training to enable them to identify, recruit, and refer other women within their close networks who meet the study’s participation criteria. Those who reported having eligible women within their networks were issued coupons designed for the study that were linked to the primary seed. Once they identified the recruits, they referred them to the central venue for the study, where they submitted the coupons that they had been issued by their study referent. The recruits were then screened for eligibility, and those who met the requirements and expressed the willingness to participate were interviewed (more details on the recruitment process are described in Zan et al. paper [30]. Overall, we completed 551 RDS interviews. Due to the comparatively high productivity in Korogocho, three primary seeds were not pursued beyond their interviews, and their related coupons were recalled.

Data were collected using a survey questionnaire programmed on Survey-CTO and password-protected handheld electronic tablets, with only authorized persons (mainly the data manager of the research team) having access to the contents. The questionnaire started with introductory questions about sociodemographic characteristics and reproductive history (number of live births, currently pregnant, current contraceptive use). We then asked questions on previous abortions and their safety, as well as the number of close female relationships aged 15–49 (with whom the respondent discusses important matters and/or with whom she shares secrets), the number of people in their networks aware of her abortion experience(s) and why the information was shared with them. The interviews were conducted either in English or Swahili, depending on the participant’s preference. All the completed data were synchronized onto APHRC’s data servers, where data management and cleaning took place by the study’s data manager in consultation with the project team.

### Data analysis

The quantitative data on the secured server were downloaded in STATA format and analyzed using STATA 14. Descriptive statistics and cross-tabulations were used to examine the respondent’s sociodemographic characteristics and their abortion experiences, including disclosure (yes and to whom), the reasons guiding the disclosure, and the safety characteristics (methods used). The abortion methods were analyzed in another article [31], and we used the same categories here: medical abortion (MA) only, manual vacuum aspiration (MVA) only, either of these methods but with innocuous methods such as drinking coke or eating honey (these three categories can be grouped as "safe methods"), unidentified pills some of which are likely MA, traditional herbs in the form of tea or ovule, and finally, known harmful methods (sharp objects, detergent, etc.)

Qualitative interviews were transcribed verbatim, and translated into English. The translation was done by one bilingual transcriber (English/Swahili speaker), and was quality checked by the team members who were native Swahili speakers and fluent in English. The team used literal translation to preserve the original meaning and intent across all transcripts. The data were analyzed through a deductive and inductive thematic analysis approach. We first developed a codebook based on the study tools and a sample of transcripts. We then coded a sample of transcripts to ensure the codes were accurate and captured any missing codes. The transcripts were then manually coded by one of the team members using the revised codebook, who regularly met with the first author to discuss challenges and get clarity and common agreement on new codes or which code to assign to some sections of transcripts.

### Ethical considerations

We sought and received approval from the AMREF Ethics and Scientific Review Committee (AMREF-ESRC P765/2020), and further received research clearance from the Kenya National Commission for Science, Technology, and Innovation (NACOSTI Ref. No: 971429). A thorough screening for eligibility was conducted with recruited women prior to the interviews to ensure details about the study were only shared with those found eligible. Further, the study was introduced as ‘Women’s Health Study’ to avoid raising suspicion and exposing study participants to the risk of confidentiality breach and intimidation at the community level. Written informed consent was also obtained from all the study participants who indicated their willingness to participate in the study. For participants who were below 18, we requested and obtained waiver of parental consent on the basis that pregnant adolescents are considered as emancipated minors in Kenya [32] and requesting parental consent would have caused risks to participants given the sensitivity of the issue of abortion and because parents were most often not informed about their abortion.

We also obtained consent to record the discussion to ensure we captured all the points during qualitative interviews. All the study participants were informed of their voluntary participation and their choice to refuse or withdraw from the study should they feel the need to do so. To further ensure privacy and confidentiality, all the interviews were conducted in a quiet and private environment free from access or being overheard by third parties.

## Results

### Socio-demographic characteristics of the qualitative study participants

For the formative qualitative study, we conducted in total 36 in-depth interviews with women aged 18–39 years. Of these, 16 were household heads, while 10 were living with their spouses. Six of the participants lived with their parents, while the remaining four lived with other relatives. Most of the participants were young women, ranging in age from 15 to 24 years with only seven being 35 years or older. In terms of educational attainment, nearly all (35) the participants had ever attended school with 22 having attended secondary level while the remaining 13 attended primary level. Majority (14) of the women were divorced/widowed/separated while another 12 were married. The remaining 10 participants were single. Most (28) of the participants had at least one child with only eight of them having none. In terms of engaging in income-generating activities, 28 of the women were either working in the informal sector as casual laborers or doing informal business while the remaining six were engaged in other income activities and only two were in school. Table 1 presents the socio-demographic characteristics of the participants in the qualitative study.

**Table 1:** Socio-demographic characteristics of the qualitative study participants.

Characteristic of participants	Participants (N = 36)
**Age group**	
15–19	7
20–29	17
30–39	12
**Highest level of school attended**	
None	1
Primary	13
Secondary	22
**Marital Status**	
Ever married	26
Single/Never married	10
**Birth history**	
At least Has a child	28
No child	8
**Occupation**	
Casual workers	18
Informal business owner	10
Student and others	8

### Socio-demographic characteristics of the RDS respondents—Quantitative survey

For the RDS survey, we obtained consent from and conducted interviews with a total of 551 women, who collectively reported 595 abortion during the reference period. Their socio-demographic information is presented in Table 2. Majority of the respondents were between the ages of 20–24 (30.3%) and 25–34 (40.8%) while close to one in ten (8.7%) were adolescent girls aged between 15–19 years. Regarding marital status, most respondents were either never married (31.9%) or were divorced/separated (44.6%). Close to 3 in 4 respondents (71.9%) were employed in the informal sector with low income and 24% were unemployed.

**Table 2:** Socio-demographic characteristics of the RDS respondents.

	Respondents (N = 551)	
Variable	N	%
**Age group at the abortion**		
15–19	48	8.7
20–24	167	30.3
25–34	225	40.8
35–49	111	20.1
**Marital status at the abortion**		
Never married	176	31.9
Married/cohabitating	104	18.9
Divorced/separated/widowed	271	49.2
**Parity at the abortion**		
Without children	106	19.2
1 to 2 children	305	55.4
3 to 7 children	140	25.4
**Education**		
No education	7	1.3
Primary	268	48.6
Secondary/higher	276	50.1
**Occupation**		
Formal work sector*	10	1.8
Informal work sector/casual workers	396	71.9
Unemployed	132	24.0
Students and others	13	2.3
**Total**	551	100

* Included manager, clerical, sales workers, formal business owner

#### What makes unwanted pregnancy and abortion a secret?

When asked about information sharing in general, participants reported sharing information within their networks based on the sensitivity and the potential impact of disclosing their secrets in their life. They further made the distinction between information that could be shared or not, and the type of confidant for each type of information. One participant explained that she usually shares only “general things to them (important friends) but for sensitive information, I keep my information secret.” Among the general information, participants listed “*issues about boyfriends*, *how boys behave*, *how ladies are frustrated in relationships and we are also trying to get those who can be with us*. *You find that most of us*, *we (girls of her age) don’t have parents*. *So we go to a friend’s home and we tell stories even up to lunchtime and we eat there*. *So*, *you leave there when satisfied*.*”* (22-year-old, secondary level education, single). On the other hand, information categorized as sensitive and secret includes getting pregnant, inducing abortion, engaging in transactional sex, or infertility. Some of the sensitive versus general information are highlighted in Table 3.

**Table 3:** General versus secret information sharing.

Age	General things shared with important friends	Secret things (never disclosed or shared only with very trusted people)
18	‘I tell them about **home**…How I am with my parents and sometimes **parents quarrel me** and when I share with them, they may give me advice’	‘The one **for abortion**. How I relate with boys…Like **having sex with a boy’**.
19	‘Just things like I **haven’t eaten**… **Sanitary pads**…’	• ‘I tell her things like **I am pregnant** and she advised me’ • ‘There is a **neighbor man who wanted me** for a relationship, I told her’
21	‘We share **issues about boyfriends**, how boys behave, how ladies are **frustrated in relationships** and we are also trying to get those who can be with us’.	• ‘Things like **getting pregnant**, she is the first person I usually tell’ • **Sex to get money**. • Sexual violence (I don’t want to tell people because that is my secret.
28	Marital issues	• ‘Maybe you’ve **found a sponsor** somewhere and **they are married’** - We both love fun. I used to love fun… We’d go dance for men and you know that there **are drugs and after that you sleep with everyone**’
28	‘I talk to her about **businesses**… Things like **alcohol and money issues’**	‘I told her about my **situation (abortion)** and asked her for advice on what to do’.
28	‘**How to find work** and if one of you has a problem…if one of us **is beaten by her husband**, others have children but **do not have survival means’**	‘We normally talk about my personal issues like **boyfriends, sickness or depression**…When he **doesn’t make you happy and he is rude**. **Sex** starts in the mind, at times, he comes when you are not interested, **he uses force to have it,** at times you get scared because **he is violent’**.
30	‘Many things like **house rents**, how at times **our businesses register loses**, so you will tell your friend that now the business seems difficult’	**‘Issues of my pregnancy** there are none of my things that she does not know’.
35	So, when we have a conflict my husband can be put down for advice and then we come together	‘**The person who impregnated me ran away**, I needed advice, so I met a friend…’
35	‘You can go to **her for help**…. If she **hears of some program**, she tells you and you go.	‘Let’s say if I have **a side-man**…Let’s say we **fight in the house with the husband**, you know men’s behaviors. …. I told her (friend), I want to **terminate this pregnancy’**.

Most women interviewed reported that they did not share information on their unintended pregnancy because “*in case you end up terminating it*, *the information can spread as people will not see the pregnancy growing*” (30-year-old, primary level education, married). Moreover, younger participants were scared of stigma and other consequences related to premarital pregnancies, such as being chased by the parents, being ostracized by the community members, or being forced to get married as explained by one of the participants:

*Even there is a friend of mine who got married while very young just because of pregnancy*. *She was alienated*.

(22-year-old, secondary level education, single)

Likewise, abortion was deemed as secret and sensitive information because of the stigma surrounding it. Abortion was reported by some participants as contemptuously being seen as an act of murder, with those who are believed to have performed abortion being named ’Muuaji’ (murderer in Kiswahili).

She is called a murderer but she wonders because she believes murderers are people who stab people and anyone who has done abortion is not a murderer.

(18-year-old, secondary level education, single).

Abusive terms like *thoroko* or *sebede* or *mtaro*—loosely translated as prostitutes—are some of the names used to refer to these women. The women who had abortion feared being associated with prostitution as it was generally considered in the community that only prostitutes do not have time to carry a child. Other names for women who had abortions were *barren* (infertile), *watu wa kutoa mimba* (people who abort), *Mama Marehemu* (mother to the dead) or *wa kujidunga* (always get and terminate). A name like *barren* was commonly used to refer to women who have had repeat abortions and are no longer able to conceive. Table 4 summarizes terminologies used to refer to women who had abortions. According to participants, almost all these names are demeaning and contribute to discriminating, alienating and shaming women and girls, as reported by one participant:

*These names make women feel uncomfortable and not worthy being in that community*, they felt *less important and that they did not “belong to the community*.

(18-year-old, secondary level education, single).

**Table 4:** Local terminologies for women who had abortion.

Terminology used to refer to women who had abortion	Meaning	Implication
Thoroko	You are a prostitute who has aborted. You are just but a prostitute	Carries stigma, as women “feel bad when called like that”
Sebede	Prostitute who abort You don’t want to carry a child to delivery so you’re a prostitute	Demeaning
Mtaro	Loose woman (promiscuous, vagina open to all men)	Demeaning
Barren	Infertile due to abortion	Bad and shameful
Mama Marehemu or	Mother to the dead or deceased	Demeaning and embarrassing
Mama kauwa	Woman who has done many abortions	Demeaning
Mutupatupana	love pleasure but she doesn’t want to give birth	Demeaning
Kutoa toa mimba or wa kutoa mimba	people who abort	Demeaning
Wa kujidunga	you always get and terminate	Demeaning
Ulienda kijitibu	Went to get treated (aborted)	Demeaning

Therefore, people tend to distance themselves from women who have had an abortion. Participants reported situations where some parents caution their children from interacting with such women citing the likelihood of their children being murdered or influenced to consider having an abortion. For instance, women from the Luo community strongly believed that those who have had an abortion are cursed and may cause young children to die if allowed to get close to or carry them. Such beliefs make women who have aborted feel guilty, isolated, and less supported. One of the participants decided to hide in a house to avoid people’s sight because she feared their reactions:

*I couldn’t come out of the house because people were talking*. *A person with a child could not come near me*, *they told their children to keep away because they would be killed*. *You know in Luo culture*, *when someone’s child has passed on they should not carry other peoples’ children*.*… Many people say that when you abort*, *it is like you are cursed*.

(19-year-old, secondary level education, single)

Although abortion is seen by the participants as something “okay to do” in circumstances such as rape, being disowned by their partner, unemployment and financial constraints, fearing of parents and community reactions, they also described it as a very shameful “thing” that women fear disclosing.

Being infertile or having pregnancy challenges in marriage was also seen as sensitive information that could expose women to stigma when known in the community.

*Such a woman is really abused and belittled*, *maybe she gets a trusted friend whom she can confide in and tell her fertility challenge*. *But most women would just dismiss you and insult you*. *Here in the community*, *abuse is just like greetings*. *People abuse each other casually and very easily*. *(…) they will tell you*, *you lady*, *you aborted and that is why you don’t have children*.

(21-year-old, secondary level education)

As explained by this participant, failing to get pregnant when married was seen as a consequence of induced abortion, hence, justifying the stigma towards women suffering from it. For all those reasons, abortion and unintended pregnancy were deemed secret information that could only be shared with a certain category of people.

#### Persons with whom the abortion information is usually shared

By construct, all the women interviewed in the RDS had their abortion known to at least another person who recruited them. When asked whether they directly shared the information regarding their unwanted pregnancy and abortion with anyone in their network, for the majority of the abortions **(**81.5%), the RDS respondents responded yes (Table 5). Among these respondents, close to half (44.7%) disclosed to only one person, while 50.9% shared the information with two or three persons, and 4.3% with more than four persons, with the highest number of confidants being 10. In total, 485 abortion seekers shared the information with 919 confidants.

**Table 5:** Number and relationship to persons to whom abortions was disclosed.

Persons informed	n	%
**Abortions with shared information**	**485** *	**81.5**
**Number of persons informed (grouped)**		
1	217	44.7
2–3	247	50.9
4+	21	1.3
**Relationship to person with whom abortions have been disclosed to**		
Friends	562	61.2
Sister/cousin/niece	208	22.6
Mother/ aunt/ grandmother	115	12.5
Partner/husband	13	1.4
Neighbor/stranger/other	21	2.3
**Total number of close relations informed about the respondent’s abortion**	**919**	

*Out of 595 total abortions (including participants who had more than one abortion)

Note that 18.5% of abortions were not shared with anyone. In the qualitative study, the main reason given for not sharing the secret was the fact that the information on unplanned pregnancy and abortion was too sensitive to be shared (see previous section).

Regarding the confidant(s), our findings (Table 5) show that most are friends (61.2%), followed by relatives such as their sister, cousin, or niece (22.6%) and mother/aunts/grand (12.5%). Finally, partners are seldom mentioned (1.4%).

#### Reasons for sharing

The qualitative findings further enlightened the relationship that participants had with their confidants. These show that participants disclosed their unwanted pregnancy or abortion information to people with whom they had an “intimate” and “trusted” relationship. Friends in whom they confided were mostly those considered as “best friends”, “trusted friends” or “very close friends’’:

*She is my friend because I find that we do everything the same*, *and that I do*, *she also does*. *Like when we meet*, *we just say let us go look for money*, *we go to the clubs and bars*. *So*, *we just maintain it as a secret*, *nobody else knows*.

(22-year-old, secondary level education, single)

*I trust X [my best friend] and if I tell her anything*, *she cannot disclose to anyone and I have never heard her telling anybody what we have discussed*, *we are still close friends even up to date*.

(38-year-old, primary level education, married).

As highlighted in the quotes, the “best” and “true” are grounded on experiences sharing secrets reciprocally and not getting them leaked, or having the same experiences or lifestyle (homophily). As explained by one participant, knowing who is the “best friend” for sharing secrets involves “studying” the person over time and events:

*Before you tell your problem*, *you must have studied them carefully*, *and when you share with them something*, *you will be assured not to hear it anywhere else*. *Even if you do something bad*, *they will tell you that you did a wrong thing here and it was not good*. *So that is what a true friend is*, *but others will lie to you and they disclose it*, *there are friends who when you share with them the secrets of your house*, *like for example*, *I told my husband this and this*, *they will tell you don’t do that again*, *you are destroying your own house*. *So you find truth in all they tell you*, *that is why*, *I became closer to them*, *in that when I go astray*, *they will warn me*

(35-year-old, primary level education, married).

Being assured that a friend had not leaked previous secrets and getting support would give most participants the courage to open up to those friends when they faced an unintended pregnancy or abortion without fear of being judged or having the information leaked. However, some participants reported that they could not share abortion or unintended pregnancy-related secrets with their church “best friends” because they will *“insist that you keep the pregnancy and be judgmental… No one from the church would let you abort*. *That is selling yourself out*. (30-years-old, primary level education, married, informal business).

Apart from friends, some participants confided in their female relatives such as sisters, cousins, aunts, or mothers based on the presumption that a family member could not betray them but will be supportive through the process. For instance, one participant who confided in her mother explained that a parent will never leak the information because they are often involved in the process and would like to protect their family’s reputation:

*If a parent knows that their girl aborted they cannot disclose it to any other person because sometimes it is them who has sponsored the abortion*.

(21-years-old, secondary level education, single).

Besides relatives and trusted/close friends, some qualitative participants also mentioned their partners among their confidants. As the person responsible for the pregnancy, participants considered it obvious that the partner should be informed to accompany them in the decision on the way forward with the pregnancy and offer them the support they may need (though in some cases the partner ended up denying the pregnancy or disappeared after the information was shared with him). It is important to note that while sharing the information with the partners seemed obvious to some participants, very few participants reported sharing the information with their partners, also revealed by the quantitative data (Table 5 above)

While most participants confided in intimate and very closed relationships in their network, few participants reported sharing the information on their unintended pregnancy and abortion with other persons such as neighbors, old friends, untrusted friends, and colleagues, among others. One of the participants explained why she informed an old friend she was not close to:

*“I called over the phone*, *she [old friend] once experienced such problems so I was looking for advice and she explained to me how to get it done*. *(…) She gave me some herbal drugs mixed with things that look like tea leaves and then I drank it for three days*, *I was bleeding and discharging clotted blood*, *the bleeding didn’t stop*, *then I went to the hospital*.

(31-year-old, secondary level education, married)

They confided in such people mainly to get support in terminating the pregnancy because they were aware that the person had previously gone through a similar journey (either because the information leaked or the person confided in them) and could have information on how and where to abort, or can provide financial support. More importantly, young women and adolescents reported that they sometimes choose to confide secrets (including unplanned pregnancies and abortion) to older women on the assumption that they have experiences on those issues and can be of good help.

*I don’t go to them to know their secrets*. *I go to them because they passed these stages*, *which I cannot go and ask my mother*, *I am not that close to them*. *That is why I prefer to have friends who are older than me*. *When I have an issue*, *they will tell me*, *they passed through the issues and she did this and this*.

(21-years-old, secondary level education, single).

Regarding the reasons that guided participants to share the information with people in their network (whether the ties were intimate or weak), Table 6 describes the main reasons reported. These included getting help in the abortion process, getting emotional support, being used to sharing their secrets mutually, and because the person told them about their abortion. Several reasons could be mentioned for the same confidant. We further cross-tabulated the people with whom abortion information was shared with the reasons for sharing to find out potential variations depending on the type of confidants (Table 7).

**Table 6:** Reasons for sharing the abortion information with the relations informed.

Variables	n (= 919)	%
**Help in the abortion (process related)**	650	70.7
**Emotional support when I had the abortion**	714	77.7
**Close ties: share close information mutually**	866	94.0
**They told me about their abortion (reciprocity)**	442	48.1
**Other reasons**	15	1.6

**Table 7:** Reasons for sharing the information by type of relationship of close relations with whom information has been shared*.

Variables	n	%
**Mother/ Aunt/ Grandmother**		
Help in the abortion process	72	62.6
Emotional support when I had the abortion	98	85.2
Share close information with each other (close ties)	112	97.4
They told me about their abortion (reciprocity)	72	62.6
Other reasons	2	1.7
Any reasons	**115**	
**Sister, Cousin, Niece**		
Help in the abortion process	173	83.2
Emotional support when I had the abortion	191	91.8
Share close information with each other (close ties)	202	97.1
They told me about their abortion (reciprocity)	137	65.9
Any reasons	**208**	
**Friends**		
Help in the abortion process	390	69.4
Emotional support when I had the abortion	412	73.3
Share close information with each other (close ties)	526	93.6
They told me about their abortion (reciprocity)	222	39.5
Other reasons	5	0.9
Any reason	**562**	

*: A single abortion may be disclosed to many close relations for many reasons

Among the women who shared the information, overall 94% reported that they did so because they maintained tight ties with their confidants that involved mutual sharing of sensitive information (Table 6). The same reason was cited by almost all participants whatever the type of confidante (from 94% to 100% according to type of tie) (Table 7). As described earlier and in the quote below, these participants had long-standing experiences of sharing mutual secrets, and supporting each other, without leaking the secrets:

*I tell her all my secrets and everything and she also tells me hers*. *We share all the information*. *Even if something happens now*, *I won’t sleep without telling her*, *I will have to go and look for her and tell her*. *(…) She is my friend because I find that we do everything the same*, *and that I do*, *she also does*. *Like when we meet*, *we just say let us go look for money*, *we go to the clubs and bars*.

(22-year-old, secondary level education, single)

This experience gave most participants the confidence to open up to friends when they found out that they were pregnant, and were contemplating abortion, during the process, or afterward without fear of being shamed, stigmatized, or reported to the community or police.

Confiding in others about their abortion was also and often guided by a need for support in the abortion process. About three-quarters of the disclosures were related to seeking help in their abortion process, either emotional support (77%) or practical help (70.7%). Support (emotional and practical) was more often cited for sisters/ cousins than for friends (Table 7). Note that mothers/aunts were somewhat less often mentioned for practical support, probably because access to abortion services is fluctuating, and better known to the younger generation. Support was almost always mentioned when disclosing to partners, and seldom for other relations (Tables 6 and 7).

Regarding the need for practical support, as said, 70.7% of the confidantes were concerned by this reason for disclosure. From the qualitative interviews, some participants explained that upon discovering that they were pregnant, they would confide in some people in their network to find out how and where to terminate the pregnancy. This participant informed her neighbor for assistance when she wanted to procure an abortion:

*I heard from people that she [neighbor] committed abortion so I went to her and forced her to give me information*, *I begged her if she could take me so that I can go there*.

(21-year-old, secondary level education, single)

In many instances, participants did not know the methods they could use to terminate the pregnancy. Therefore, they had to seek the information, which required talking to someone. As highlighted in the quote, trusted friends were key in supporting participants to obtain the relevant information for their abortion. For instance, in addition to being her trusted friend, the participant above (like others) also knew that her friend had undergone an abortion in the past and was still alive, which meant the friend would be of help in getting information on the ‘right’ method to use. Moreover, the participants who already had some information on methods to terminate a pregnancy, but were not sure about the safety or could not afford the cost would talk to people in their network to get additional information on safer or cheaper methods, as highlighted in the following quote:

*The secret one [the friend] is already aware and so I am just seeking more opinion so that I get the best idea*. *Somebody will tell you about tea leaves*, *somebody tells you about soda for dilution*, *another person says go to the chemist*, *there are tools there*.

(19-year-old, secondary level education, single)

In addition to getting information on methods for aborting, some participants reported confiding in trusted people because they needed financial support to perform their abortions. Partners and most relatives were often confided in because the participants needed money to pay for the drug or the services of the providers:

*I was 16 years*, *I went and hid in that boy’s place*, *it was far away in Kibera and he supported me and gave me even money*. *He treated me well and after aborting I stayed and he began looking down upon me*, *he was saying that he could not live with one who has aborted*, *and many other things*. *Remember he is the one who supported you and has money for abortion but just changed*. *I can’t live with a person who has rotted her seeds*, *He could say you’re old*. *He goes out and comes home with his issues that she has someone who will give birth to her children So*, *I just thought instead of staying here let me go back and stay with my father as usual*.

(22-year-old, secondary level education, single)

While their attitudes toward their female partners may shift, occasionally resulting in relationship breakdowns, interviews with girls and women indicate that their partners played a pivotal role in both the decision-making process and the access to abortion services. According to participants, they often had the right network to find the person to perform the abortion and had the resources to pay for it. Many of the participants were young, jobless, or actively working for meager wages. Therefore, they could not afford abortion services, especially from safe procedures from trained providers or pharmacists. Hence, they needed financial support from their partners or relatives to procure their abortion. In the case of this girl, the partner also assisted with the abortion process to avoid her parents knowing that she was pregnant and trying to procure an abortion.

Regarding the emotional support (reported by 77% of the RDS respondents, Table 6), the qualitative participants further explained that the abortion-seeking process was very emotionally laden and lonely due to the stigma described above. Participants explained that upon discovering they had an unintended pregnancy, they often battle with the stress of deciding whether to keep the pregnancy, the internalized stigma resulting from the feeling of breaching social norms. When seeking abortion services, they also battle with the uncertainty associated with the process, the fear of facing complications such as death (stories common in their neighborhood), and the fear of people finding out that they are procuring an abortion. Lastly, they may also be confronted with emotional distress after the abortion procedure because of internalized stigma. For all these reasons, some participants would confide in their best and trusted friends, their trusted relatives, or their partners to lean on them in the process and lift the emotional burden.

Lastly, women also confided in some intimate relationships as well as weak relationships (as mentioned above) because they knew the person also had an abortion. This was the case for 48.1% of the RDS respondents (Table 6). Because their confidant had earlier confided in them about an abortion, they then had the courage to inform the confidant about their abortion, either at the time of the procedure or afterward. They trusted that their confidant (who had a similar experience previously) would not judge them, as highlighted by this participant:

*I then went to my friend (name) “I want to terminate this pregnancy”*. *She asked me why*? *I told her why do you ask me yet you also recently aborted because of some challenges*? *I reminded her*. *She told me she will take me where she aborted hers*. *She is a good woman*. *She will give you here and when you get to the house*, *take like five glasses*, *and be cool*, *don’t even bathe*. *If you bathe*, *use hot water*, *not cold one*.

(35-year-old, primary level education, married)

Recalling to her friend that she also terminated a pregnancy, ensured that the friend does not discourage nor judge her. Sharing the information with such confidants was also guided by the assurance that they would be of help in seeking services and in getting emotional support.

Some women did not deliberately share the information regarding their abortion, but this rather happened against their will. In some situations, the abortion was disclosed because of complications. For instance, Marie, a 25-year-old divorced woman, tried to abort a four-month pregnancy using tea leaves and undiluted juice that she once heard friends talking about. This led to heavy bleeding for two days. Feeling unconscious and afraid of dying, she requested her neighbor to take her to the hospital for care. There, the neighbor discovered that Marie had induced her abortion, and when back home, she informed three of her neighbors:

*I was bleeding and in the process I called her [my neighbor] to take me to the hospital*. *I told her to accompany me because the rest were not around and I was feeling unconscious*. *She is the one who came and disclosed this information to people*.*(…) I had taken tea leaves and undiluted juice*, *I was now bleeding*, *so I went to a provider to completely terminate the pregnancy*.

(25-year-old, primary level education, divorced)

Because this neighbor was not in an intimate and trusted relationship, the information on the participant’s abortion ended up being spread within her neighborhood. If this participant had the courage of remaining within her community, others, like the participant in the quote below were forced to relocate after the information on her abortion was leaked to the public because of severe complications:

*The pregnancy grew up to the seventh month*. *We could collect money bit by bit and later we went to a cheap place for abortion*. *… He poorly aborted my pregnancy and I was crying loudly*. *It was meant to be secret but everybody became aware*. *He did it very badly*. *I was taken to X (referral hospital)l when I was unconscious like a dead person*. *People talked very badly*, *‘just die’*. *Even doctors were not concerned with you*. *I could say to the doctor*, *please help me*, *I am dying*, *they replied just die*. *I cannot go to X (referral hospital) again*. *I Would rather Just die*. *…I wanted to keep it secret*. *I didn’t want it to be known in the community and but it happened*. *That is why I left*. *If someone decides to spread it there in Kibera*, *who knows me over there*? *Nobody*.

(22-year-old, secondary level education, single)

In her new neighborhood, only her close friend is now aware, and providing her with emotional and financial support.

#### Relationship between the confidant and type of method used

We also considered the link between the type of confidant and the method the woman ended up using to terminate the pregnancy. For Table 8, we narrowed down to those who reported sharing the information to get help in terminating their pregnancy to see the potential link between the type of confidant and method used. As noted in the preceding table, a single abortion may be disclosed to several confidants, and the unit of analysis here is the episode of disclosure to a relation. After cross-analyzing the responses of respondents who reported that they shared their abortion to seek help in the abortion process with the method used, the findings show that unidentified pills, some potentially medical abortion (MA) was the most commonly used method, across all types of confidantes. About 53.8% of respondent who disclosed their abortions to their partner/husband relied on this method, 52.4% of those who confided to another relation, 47.2% to a friend and 46.7% to sister/cousin. Unidentified pills were however somewhat less often used in those abortions where mothers/aunts/grandmothers were informed (37%). Traditional methods (herbs, concoctions, ovules ingested) were also popular, and were predominantly found among those abortions disclosed to mother or aunt (33%), and somewhat more rarely in abortions shared with sister or cousin (23%), friends (20.1%), neighbors (19.1%), and partner or husband (15.4%). Known harmful methods were also relatively common, and especially often mentioned in cases where help was sought from neighbors and other weaker ties (28.6%), and somewhat less often in the other instances of disclosures: partner (23.1%), sister or cousin (20.2%), mothers/aunts/grandmothers (17%), friends (16.6%). Safe methods, such as medical abortion drugs or manual vacuum aspiration (MVA) (with or without innocuous methods like coffee, beer, etc.) were rarely used, whatever the type of relationships contacted, but with a gradient. Indeed 14.4% of the abortions disclosed to mothers/aunts/grandmothers relied on one of these safe methods, 10.3% of abortions shared with friends, 9.2% of the abortions confided to sister or cousin, 7.7% of those shared with the partner, and none of the abortions where another contact was involved.

**Table 8:** Distribution (number and %) of method used by type of confidant informed.

	MA Only (n %)	MVA only (n %)	MA/MVA with innocuous methods	Unidentified pills with/out safe or innocuous method	Innocuous beverages and foods only (%)	Traditional/herbal preparations/ovules ingested (n %)	Any use of known harmful methods (n %)	Total number of close relations informed
**Mother Aunt Grandmother**	10 (8.7)	4(3.5)	0(0)	43(37)	0(0)	38 (33.0)	20(17)	115
**Sister, Cousin, Niece**	18 (8.6)	0 (0)	1(0.5)	99(47.6)	0(0)	48(23.1)	42(20.2	208
**Partner/Husband**	0 (0)	0 (0)	1(7.7)	7(53.8)	0(0)	2(15.4)	3(23.1)	13
**Friend**	19 (3.4)	51 (9.1)	5(0.9)	265(47.2)	16(2.8)	113(20.1)	93(16.6)	562
**Neighbor/ Work colleague/ Strangers**	0 (0)	0 (0)	0(0)	11(52.4)	0(0)	4(19.1)	6(28.6)	21

The qualitative findings show that participants were advised on different methods by their friends or relatives. This included herbs or medical abortion pills as explained by the following participant:

*She [the friend] told me that we will look for money so that we buy pills*, *there are abortion pills that ladies use*, *it is 3000 (KES)*.

(22-year-old, secondary level education, single).

The results also show that weaker ties were involved when accessing more dangerous (and cheaper) methods. In the case of this participant she could not afford the KES 3000 (~US $27 then), and instead she visited a quack that she “heard people in the community talking about”. The provider performed a surgical procedure that ended up into a severe complication: “I really wailed, I reached X hospital when carried by seven mothers.”

Some confidantes took participants to providers they knew (some of whom were using unsafe procedures), or advised them to use herbs such as tea leaves.

*I: What did she tell you to do or where did she direct you to go to?*

*R*: *At first*, *she told me to use tea leaves and if it doesn’t work*, *I should go to my grandmother*.

(18-year-old, secondary level education, single)

As highlighted in the quotes, this participant followed her friend’s advice and was able to terminate the pregnancy using tea leaves.

## Discussion

This study provides interesting insights on the dynamics around the disclosure of abortion among adolescents and young women in informal settlements in Kenya. In navigating daily life, social crises, and their reproductive health, women and girls who participated in this study confide and seek support through their close, best and intimate relationships, namely friends, church-mate, neighbors or their relatives. Our findings showed how the secret sharing fits into pre-existing dynamics and codes and mutual support within women networks. Disclosure behavior among women was found to be determined by several factors such as the sensitivity of the information, the level of familiarity, and most importantly, trust. Women displayed a heightened awareness of the sensitive nature of the information at hand, and the potential ramifications of divulging it. This aligns with Simmel’s observations that the choice of whom to confide in heavily is significantly influenced by the type of information being shared, and the impact it may have on the relationship [24].

Consequently, our findings show that women may not have disclosed their abortions because they perceived it to be a highly stigmatizing occurrence that would bring about personal shame and discrediting. Similarly, other studies done in Kenya suggest that women often restrain from disclosing their abortion as a way of curbing incidences of stigma in the community [33]. Moreover, the current study indicates that some women may have internalized the stigma, manifesting in feelings of self-blame and shame, further enhancing the need for secrecy. These results echo those of Astbury-Ward et al. [34] on perceptions of women who have had an abortion in England and Wales, where stigma among the women manifested in self-blame, shame and eventually secrecy. Combined, these findings highlight the need for future research around the complexity surrounding the social and personal dimensions of stigma that influence women’s disclosure decisions and how it can affect women’s likelihood of accessing safe services. Linking our findings to the systematic review conducted by Rossier et al. [23] we found that women and girls in Korogocho and Viwandani often fall under either of these two categories: “Women overwhelmingly need help from personal contacts to find services and can confide in trusted contacts in order to find services (high stigma) or "Women overwhelmingly need help from personal contacts to find services but cannot disclose to contacts" (hyper stigma) [23]. Indeed, access to safe abortion services are often hidden because of the legal restriction and stigma, and the access requires opening up to someone who has the information, some women may end up using unsafe procedures because they could not confide in someone and get the right guidance. Though abortion services are clandestinely available, the access is often subjected to women having information on these methods and where to find it, as well as the resources to afford it. In both cases, women often have to disclose to someone in their network to find the information (especially those who do not have access to the internet for instance) or to get money to purchase or pay for the services [23]. Most often, the quest for social safety because of the high stigma may push women and girls to seek abortion services in secret and prioritize methods that can be accessed discreetly on their own, without the help of their community or network [35].

When women disclosed their abortion, our study shows that a majority of women disclosed their abortion to their friends, while their partners were seldom mentioned. These findings contradict some of the previous studies done in Kenya, such as Osur et al. [22] whose mixed method study in rural Siaya, showed the man responsible for the pregnancy as the primary recipient of the news, followed closely by friends. One possible explanation for this could be that women living in urban areas of Kenya may be engaged in casual or transactional relationships, which does not qualify their sexual partners for becoming confidant unlike their rural counterparts who are often married. On the other hand, urban friends are suitable for sharing secrets because they have similar experiences (i.e. transactional sex, unintended pregnancy and abortion), and can therefore provide them with information on abortion methods as shown in other studies in Kenya as well [36]. As a result, they may feel more comfortable discussing the sensitive topic of abortion while seeking out methods to use.

Indeed, our findings show that the main reason why women choose to disclose their abortion to others was not just because of having close ties, where women share information mutually, but also because of the need for support (emotional or practical). These results were in tandem with findings from a systematic review on the trajectories of women’s abortion-related care, where disclosing the information, was mainly driven by the need for support (monetary, (mis)information, emotional), while disclosing to the wrong party may result in negative consequences [20]. The implications of intentional and unintentional disclosure, as we found in our study, translate to women facing judgment and abuse. Women in turn fear the implications of disclosing their pregnancies as well as their intentions to terminate, therefore delaying initiating the abortion process [37]. This culture of secrecy around mistimed pregnancies and abortion has a profound impact: it inhibits women from seeking professional reproductive health information and care, putting them at risk for complications, morbidities, and death [38].

Our findings also show that most participants, who confided to seek help in terminating (finding a method or obtaining financial support) were directed towards unidentified drugs, traditional or known harmful methods. This implies either the low circulation of information on safer methods within the community (more pronounced among the older generations) and/ or widely shared challenges in accessing safe methods as shown in the qualitative findings as well as in other studies in Africa [39–41]. Other studies have also demonstrated how the lack of knowledge of the women intermediaries and confidants on safe methods can lead to them using unsafe procedures [42].

## Conclusion

Our findings show that the disclosure of information related to is a complex process that varies from a woman to another and is embedded in existing codes regarding the circulation of information on intimate and sensitive issues as well as “help” seeking behaviors. In a context where abortion is restricted and safe abortion and related services are hidden (i.e. psychosocial support), accessing abortion methods or emotional support requires opening up to someone in women and girls networks. While participants may be able to overcome the fear of stigma and confide in friends, female relatives, their partners or neighbors to get help in securing their abortion, the lack of information on the safe methods to terminate a pregnancy adding to the lack of financial resources, often lead to them being geared towards unsafe procedures. The findings suggest the need for multilevel intervention to reduce the abortion stigma within the community and improve the access to information on safe abortion procedures. Future research could explore how strengthening the circulation of information on safe methods within communities, using community champions and intermediaries (i.e. peers, mothers, and partners) is effective in increasing the likelihood of women and girls being directed to safe methods to enhance their use to reduce the complication that may come from unsafe procedures.
